# Anatomical heterogeneity of tendon: Fascicular and interfascicular tendon compartments have distinct proteomic composition

**DOI:** 10.1038/srep20455

**Published:** 2016-02-04

**Authors:** Chavaunne T. Thorpe, Mandy J. Peffers, Deborah Simpson, Elizabeth Halliwell, Hazel R. C. Screen, Peter D. Clegg

**Affiliations:** 1 Institute of Bioengineering, School of Engineering and Materials Science, Queen Mary University of London, Mile End Road, London, E1 4NS, UK; 2 Department of Musculoskeletal Biology, Institute of Ageing and Chronic Disease, University of Liverpool, Leahurst Campus, Neston, CH64 7TE, UK; 3 Centre for Proteome Research, Institute of Integrative Biology, University of Liverpool, Crown Street, Liverpool, L69 7ZB, UK

## Abstract

Tendon is a simple aligned fibre composite, consisting of collagen-rich fascicles surrounded by a softer interfascicular matrix (IFM). The composition and interactions between these material phases are fundamental in ensuring tissue mechanics meet functional requirements. However the IFM is poorly defined, therefore tendon structure-function relationships are incompletely understood. We hypothesised that the IFM has a more complex proteome, with faster turnover than the fascicular matrix (FM). Using laser-capture microdissection and mass spectrometry, we demonstrate that the IFM contains more proteins, and that many proteins show differential abundance between matrix phases. The IFM contained more protein fragments (neopeptides), indicating greater matrix degradation in this compartment, which may act to maintain healthy tendon structure. Protein abundance did not alter with ageing, but neopeptide numbers decreased in the aged IFM, indicating decreased turnover which may contribute to age-related tendon injury. These data provide important insights into how differences in tendon composition and turnover contribute to tendon structure-function relationships and the effects of ageing.

Tendons are an integral component of the musculoskeletal system, transferring muscle-generated force to move the skeleton. For this function, they require high uniaxial strength, which is provided by the abundant type I collagen molecules, hierarchically arranged and aligned in the direction of force transmission. Specific tendons have an additional function, stretching and recoiling with each stride to store and return energy, which decreases the energetic cost of locomotion. In addition to high uniaxial strength, energy storing tendons also require a degree of compliance for efficient energy storage, experiencing much higher strains than tendons that are purely positional in function^1^,^2^. Our previous data indicate that the extra extensibility required by energy storing tendons is facilitated by sliding between the collagen-rich fascicles, which are the largest subunits of tendon. This sliding behaviour is governed by the interfascicular matrix (IFM, also referred to as the endotenon), which binds adjacent fascicles together. While data indicate that the IFM is crucial for efficient function of energy storing tendons, the composition and structure of this matrix remains poorly defined, such that IFM, fascicle and resulting whole tendon structure-function relationships are not well understood.

Tendon provides an ideal system to investigate tissue structure-function relationships as it is a relatively simple fibre composite material, consisting of two distinct compartments of differing composition, often referred to as material phases. Establishing the compositional differences between phases helps provide insight into how they interact to give rise to individual tissue mechanics. Establishing these relationships in a simpler system such as tendon may therefore be of relevance to understanding structure-function relationships in more complex loaded tissues.

However, little work had been undertaken to assess the tendon proteome until very recently, with studies characterising alterations in tendon protein profile during development^3^, with ageing^4^ and in disease^3^,^4^, as well as differences between tendons and ligaments^5^ and at the musculo-tendinous junction^6^. However, no comparison has been made of the proteome of different anatomical compartments of the tendon tissue proper. The few studies that have investigated IFM composition using other techniques have identified proteins including elastic fibres^7^,^8^, proteoglycans^9^,^10^,^11^ and collagens^10^,^12^,^13^. Previous data indicate that as well as the IFM having a different composition and structure, the turnover of this matrix also differs, occurring more rapidly than in the fascicular matrix (FM)^14^,^15^.

It has also been demonstrated that, while increasing age causes minor alterations in fascicle mechanical properties, much larger age-related changes are seen in the IFM, with increased stiffness and decreased elasticity in the IFM of energy storing tendons^16^,^17^. These data suggest that the structure of the IFM is altered with ageing in energy storing tendons specifically, which may explain why aged tendons are more prone to injury^18^,^19^. While previous data have demonstrated distinct proteomic profiles in young and old tendon, with alterations in the levels of proteins involved in matrix organization and regulation of cell tension^4^, no previous studies have assessed how IFM composition is altered with ageing, such that the mechanisms that result in reduced elasticity in aged tendons remain poorly understood.

In this study, we assessed the proteomic profile of the FM and IFM and identified age-related changes in protein content and extracellular matrix degradation in equine tendon tissue. The horse is an accepted and relevant model in which to study musculoskeletal ageing, as it is a relatively long-lived, athletic species in which age-related musculoskeletal diseases, including tendon injury, show a very similar epidemiology, aetiology, and pathology to that seen in human age-related musculoskeletal diseases^19^,^20^,^21^,^22^. In both species the most commonly injured tendons are those that store and return energy during locomotion. These are the Achilles tendon in the human^23^, and the superficial digital flexor tendon (SDFT) in the horse, which is one of the most extreme examples of an energy storing tendon^24^. We hypothesised that, in the equine SDFT, the IFM would have a more complex proteome than that of the FM, with a greater number of cellular proteins and more rapid turnover, as determined by identification of cleavage of matrix molecules. We additionally hypothesised that age-related alterations would be greater in the IFM than in the FM. To test this hypothesis, we isolated regions of IFM and FM from the SDFT from young and old horses using laser-capture microdissection (LCM), and assessed protein profile using label-free relative quantification to identify differentially abundant proteins between tendon regions and age groups. Further, we assessed fragmentation patterns of specific proteins by identifying neopeptides to give an indication of matrix turnover.

## Results

### Protein identification and ontology

Following LC-MS/MS separation of laser captured samples, peptides and corresponding proteins were identified using Peaks® 7 PTM software (Version 6, Bioinformatics Solutions, Waterloo, Canada). In young samples, 130 and 92 proteins were identified in the IFM and FM respectively. The number of proteins identified in old tendon were similar, with 123 in the IFM and 89 in the FM. Supplementary Tables 1 and 2 provide detailed information on the identification of peptides mapped to each protein. The datasets were transformed to non-redundant gene identifier lists of the respective human homologues and subjected to gene ontology using MatrisomeDB to classify extracellular matrix (ECM) proteins. Proteins that were not classified as part of the matrisome were subjected to further classification using Protein ANalysis THrough Evolutionary Relationships (PANTHER) classification system. Protein-protein interactions were mapped using the Search Tool for Retrieval of Interacting Genes/Proteins (STRING) (Supplementary Fig. 1). Protein classifications are shown in Table 1.

16 collagen chains were identified in the IFM, and 15 in the FM, these related to 10 members of the collagen superfamily. Of these, 8 were common to both the IFM and FM (see Table 1). Col7A1 and Col22A1 were only identified in the IFM, whereas Col17A1 and Col18A1 were only present in the FM. Eight proteoglycans were identified in the IFM, and 7 in the FM; only heparin sulphate proteoglycan 2 was identified in the IFM and not in the FM. 13 glycoproteins were identified in the IFM, and 8 in the FM; 5 of these were found in both the IFM and FM. Adiponectin, 3 laminin subunits, periostin, TGF-β interacting protein, tenascin-x, and dermatopontin were only found in the IFM, whereas fibrillin-2, thrombospondin-1 and lactoadherin were only identified in the FM.

### Label-free relative quantification

**Figure 1:**
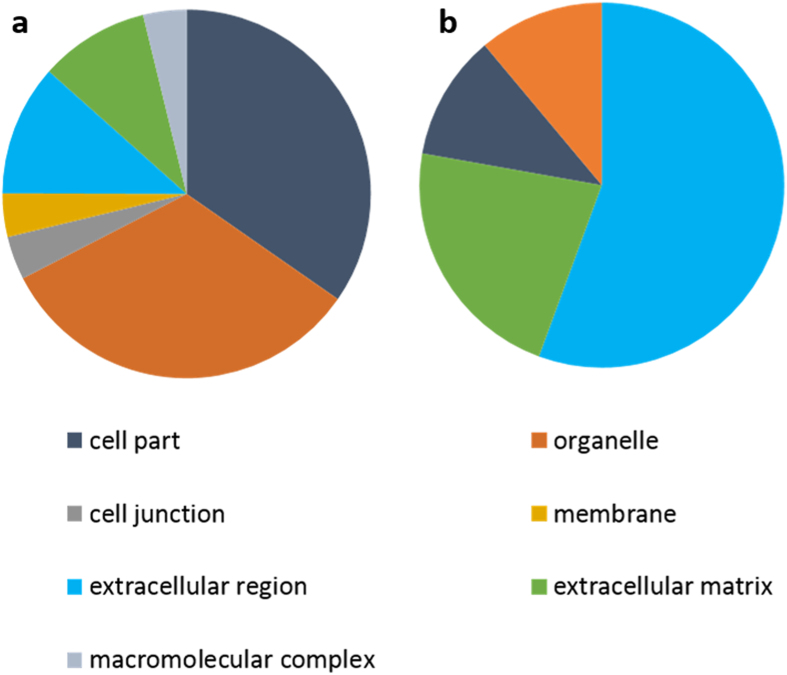
PANTHER classification was used to classify proteins that were present at higher abundance in the IFM (a) and FM (b). Note the higher abundance of cell-related proteins in the IFM, and a greater abundance of extracellular matrix proteins in the FM.

**Figure 2:**
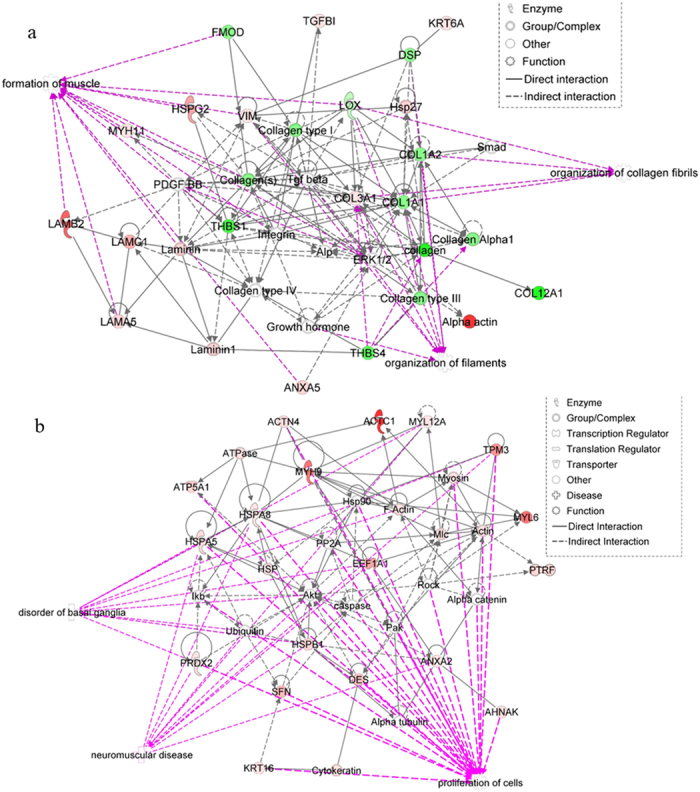
Top scoring networks derived from the proteins with different abundance in the IFM and FM. IPA identified 2 significant networks with scores of 40 related to development (**a**), and cellular movement and nervous system function (**b**). Green nodes, greater protein abundance in the FM; red nodes, greater protein abundance in the IFM; white nodes, proteins not differentially abundant between the FM and IFM. Intensity of colour is related to higher fold-change. Key to the main features in the networks is shown. Significant functions related to network 1 included muscle formation, organisation of collagen fibrils and organisation of filaments (**a**, p < 0.0001). Diseases and functions related to network 2 included cell proliferation, neuromuscular disease and basal ganglia disorders (**b**, p < 0.0001).

Relative protein abundance was assessed by label-free relative quantification (Progenesis QI^TM^ software, Version 4, Waters, Manchester, UK). Progenesis analysis revealed greater than 2-fold differential abundance of 92 proteins (60 with ≥2 peptides) between the IFM and FM in young tendon. Of these proteins, 73 (48 with ≥2 peptides) had higher levels of abundance in the IFM, and 19 (12 with ≥2 peptides) were more abundant in the FM (Table 2; Supplementary Table 3). According to Matrisome DB and PANTHER analyses, the majority of proteins with higher abundance in the IFM were cellular proteins, whereas those with higher abundance in the FM were mainly ECM proteins (Fig. 1). ECM proteins enriched in the FM included Col1A1, Col1A2, Col12A1, thrombospondins 1 and 4, fibromodulin, cartilage oligomeric matrix protein (COMP) and decorin. Ingenuity Pathway analysis (IPA) of the differentially abundant proteins generated several networks that were enriched. According to the top scoring networks, the differentially expressed proteins between the FM and IFM were associated with development, cellular movement and nervous system function (Fig. 2). Functions associated with proteins enriched in the IFM included muscle formation, neuromuscular disease and disorders of basal ganglia (Fig. 2), while proteins enriched in the FM were associated with organisation of collagen fibrils and filaments (Fig. 2). Significant IPA canonical pathways that are upregulated in the IFM are shown in Supplementary Table 4, these include integrin-linked kinase (ILK) and actin cytoskeletal signalling.

### Effect of ageing

Results were similar in old tendon, in which 80 proteins (49 with ≥2 peptides) were more abundant in the IFM, and 19 (13 with ≥2 peptides) were present at higher levels in the FM (Supplementary Table 5). Ageing did not result in large changes to protein composition within either the IFM or FM, with 7 and 9 proteins showing differential abundance with ageing in the IFM and FM respectively (Supplementary Tables 6 and 7). Fibromodulin abundance decreased with ageing in the IFM and FM. Similar networks were identified in old tendon as those seen in young tendon (data not shown).

### Identification of ECM fragmentation patterns

Significantly more ECM neopeptides were identified in the young IFM than in the FM (571 ± 115 vs 305 ± 139; p = 0.03). The number of neopeptides decreased significantly with age in the IFM (old IFM: 191 ± 86 neopeptides; p = 0.002), but did not change with ageing in the FM (old FM: 455 ± 289 neopeptides). There was no significant difference between number of neopeptides seen in the FM and IFM from old tendons.

A summary of the number of neopeptides identified in each condition is shown in Table 3. Supplementary Table 8 contains peptide sequences for neopeptides showing differences between conditions. Very few neopeptides were identified for non-collagenous proteins. However, a large number of collagen neopeptides were present. More collagen type I, III, V and XI neopeptides were identified in the IFM than in the FM in tendons from young horses (Table 3). However, with increasing age the number of neopeptides in the IFM decreased, meaning that more collagen neopeptides were seen in the FM than in the IFM in old tendons. The number of neopeptides in the FM did not appear to be affected by ageing.

### Differences in cell content between tendon regions

**Figure 3:**
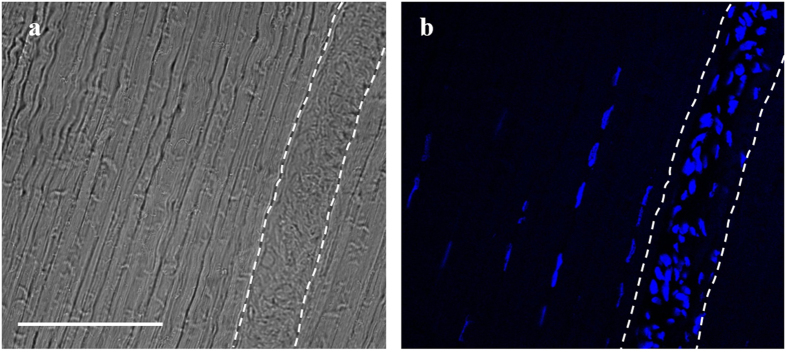
Images of longitudinal sections of the SDFT, showing tendon structure (a) and cell morphology (b). The IFM is enclosed by dashed lines. Note the greater cell number, and distinct morphology, of cells in the IFM. Scale bar = 100 μm.

Cell content was assessed by fluorescent staining of cells in tendon sections, as shown in Fig. 3. Cell number was significantly greater in the IFM, with 4139 ± 1220 cells/mm^2^, compared to 382 ± 138 cells/mm^2^ in the FM (p < 0.0001; Fig. 3). There was a trend towards decreased IFM cell number with ageing, but this was not significant (3009 ± 1155 cells/mm^2^; p = 0.06); the number of cells in the FM was unaffected by ageing.

### Validation of mass spectrometry results by Western blotting

**Figure 4:**
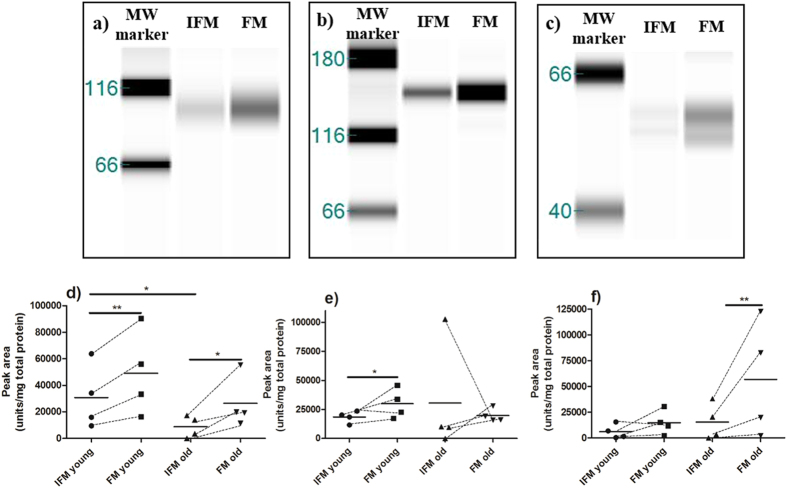
Fibromodulin (a,d), COMP (b,e) and decorin (c,f) abundance were confirmed by Western blotting. Representative Western blots for fibromodulin (**a**), COMP (**b**) and decorin (**c**). Abundance of each protein is expressed semi-quantitatively (**d**–**f**), relative to total protein content. Each symbol represents an individual sample, and lines indicate paired IFM and FM samples from the same tendon. Statistical differences were assessed between the IFM and FM using paired t-tests for normally distributed data, or Wilcoxon matched-pairs signed rank test for non-normally distributed data. To assess differences with ageing, unpaired t-tests or Mann Whitney tests were used. *p < 0.05; **p < 0.01.

Abundance of fibromodulin, COMP and decorin, as assessed by Western blotting, is shown in Fig. 4. Apparent molecular weights were larger than expected for fibromodulin and COMP^25^,^26^, this is likely because samples were not deglycosylated prior to Western blotting. In agreement with the mass spectrometry data, fibromodulin abundance was significantly (p = 0.0008, Fig. 4a,d) greater in the FM than in the IFM, and decreased significantly with ageing in the IFM (p = 0.02). There was a trend towards decreased fibromodulin abundance in the FM with ageing, but this was not significant (p = 0.09). COMP abundance was significantly higher in the FM than in the IFM in young samples (p = 0.04), also supporting the mass spectrometry findings. There was no difference in COMP abundance in the FM and IFM of aged tendons, likely due to the presence of an outlier (Fig. 4b,e). There was a trend towards higher decorin abundance in the young FM compared to the IFM (p = 0.08); this difference was significant in old samples (p = 0.02; Fig. 4c,f).

## Discussion

This is the first study to characterise differences in the protein content of different anatomical compartments of the tendon matrix, and determine how the proteome of these distinct matrices is altered with ageing. The results support our hypothesis, showing that the interfascicular and fascicular matrices have distinct protein profiles; the IFM contains many cellular proteins, but also collagens and glycoproteins, whereas the FM is dominated by collagens, with a smaller amount of non-collagenous and cellular proteins. The levels of only a few proteins were altered with ageing, but the results suggest that collagen turnover may decrease with ageing, particularly in the IFM.

While the use of laser capture microdissection allows the isolation of distinct regions of tendon tissue with minimal contamination, it only allows the collection of very small amounts of tissue, such that proteins present at low abundances may be below the detection limit of the mass spectrometry instrumentation and so cannot be identified. Detection of low abundance proteins is also made more difficult due to the high abundance of collagens and other ECM in tendon. Future studies could use hexapeptide library protein normalization^27^, which would allow identification of low abundance proteins.

When considering the number of proteins identified, a greater number were identified in the IFM than in the FM. However, a similar percentage of proteins were classified as extracellular and non-extracellular in both matrix compartments (63% ECM vs 37% non-ECM, Table 2). In total, 60 proteins were present at significantly different levels in the IFM and FM. In the FM, the majority of these proteins were ECM proteins, whereas proteins found in higher abundance in the IFM were predominantly cellular. The proteins enriched in the FM included collagen types I and XII, thrombospondins −1 and −4, COMP, fibromodulin and decorin. This is supported by a previous immunohistochemical study that has demonstrated that type I collagen and COMP are localised to the fascicular regions of the SDFT^12^. Many of these proteins are involved in collagen synthesis and regulation of fibril formation^28^. It has recently been demonstrated that fibromodulin modulates the C-terminal cross-linking of collagen molecules, and that the absence of fibromodulin results in decreased collagen fibril organisation and tendon strength^29^,^30^. Fibronectin was also present at higher levels in the FM. Fibronectin plays an important role in matrix assembly and organisation, forming a bridge between cell surface receptors and extracellular matrix components including collagens and proteoglycans^31^,^32^.

In the IFM, only 1 of the members of the collagen superfamily, collagen type III, was enriched. It has been shown previously that collagen III is localised to the IFM in the equine SDFT^12^. It is well established that collagen type III concentration increases in injured tendon^12^,^33^, and it is also essential for collagen type I fibrillogenesis^34^. In addition, 1 proteoglycan (basement membrane-specific heparan sulfate proteoglycan core protein, also known as perlecan) and 5 glycoproteins (3 laminin subunits, transforming growth factor β-induced protein and adiponectin) were enriched in the IFM. Previous studies have shown that perlecan is localised to the pericellular region of oval shaped cells in the mid-substance of the supraspinatus tendon^35^, and that perlecan and laminin are predominantly found the matrix surrounding mouse tail tendon fascicles^36^. These proteins are both involved in promotion of cell proliferation and matrix production^37^,^38^, suggesting that the IFM may be more active than the FM. Indeed, the majority of other proteins enriched in the IFM were cellular proteins, including histones, ribosomal protein and cytoskeletal proteins. While some of these differences may be due to the elevated cell content in the IFM, which is approximately 10 fold greater than in the FM, it is also possible that these differences indicate a distinct phenotype between the 2 cell populations. It is evident that cell morphology differs between the IFM and FM, with rounder cells in the IFM, whereas FM cells are more elongated, and previous work indicates that cells within the IFM are more active than their counterparts in the FM^15^. However, tendon cell populations remain largely undefined and as such this is an important area for future research.

Analysis of differential networks using IPA showed significant enrichment of pathways relating to muscle formation in the IFM. Samples were taken from the mid-metacarpal regions of the tendon, far removed from the muscle insertion, and so it is unlikely that this is due to sample contamination. A previous study of the Achilles transcriptome has shown the expression of muscle-related genes in tendon^39^ and a myogenic signature is present in healthy articular cartilage which is lost with ageing^40^, suggesting that, while these proteins are generally associated with muscle, they may play an important role in maintenance and function of other musculoskeletal tissues.

The abundance of a small number of proteins was altered with ageing. Notably, fibromodulin abundance was significantly lower in aged tendon in both the FM and IFM supporting our previous findings^4^. Fibromodulin plays an important role in modulating collagen fibrillogenesis^30^ and cross-linking^29^ and so the reduction with ageing may impede the repair of damaged collagen fibres in aged tendon. In the IFM, the majority of proteins that showed changes in abundance with ageing were cellular proteins. In the FM, collagen type VI and mimecan levels were reduced with ageing, whereas the abundance of a few cell-related proteins increased with ageing. Interestingly, a recent transcriptomic analysis of the ageing Achilles tendon revealed no alteration in the expression of genes relating to ECM proteins, but showed a significant dysregulation of gene sets related to cellular function and maintenance with increasing age^39^. However, it is possible that ageing may affect organisation of the ECM, which will not be identified by the compositional analysis performed in the current study.

Unexpectedly, lubricin and elastin were not identified in either the IFM or FM. Previous studies have shown that both of these proteins are present in tendon, and also localised to the IFM^8^,^41^, where they may play a role in facilitating sliding and recoil between fascicles. Unfortunately we conclude that we were not able to detect the presence of these proteins with our mass spectrometry methods. Sequences relating to equine lubricin are very sparse, and the Uniprot sequence for equine lubricin (Q×69×3) contains a sequence that is only 63-residues in length, therefore lubricin peptides in the samples analysed may not have been identified using the UniHorse database. It is challenging to identify elastin using mass spectrometry as elastin is highly hydrophobic and has a highly repetitive sequence^42^. Previous mass spectrometry studies of elastin have included an elastase digest step^42^,^43^, or used 2D separation techniques which could be implemented in future studies. Due to incomplete annotation of the equine proteome, it is possible that a small number of proteins may not have been identified.

The majority of neopeptides identified in this study were collagen neopeptides. Fewer neopeptides for non-collagenous proteins were identified than in our previous study on tendon fascicles^4^, this may be attributed to the lower amount of material obtained from laser capture microdissection. While a small number of neopeptides were identified for proteoglycans, none of these neopeptides showed differences between tendon compartments or with ageing (data not shown). It is possible that some of the neopeptides identified were generated post-mortem during tissue processing as cell death can release intracellular proteases, but protease activity was minimised during processing by chilling and rapid post-mortem dissection of the limbs and snap-freezing of tissue.

When considering differences in neopeptide numbers between groups, more collagen-related neopeptides were identified in the IFM than in the FM, despite the greater abundance of several collagens in the FM, suggesting that collagens in the IFM may be turned over more rapidly than in the FM. This is supported by previous data showing greater levels of the collagenases MMP-1 and −13, and the collagen degradation marker C1, 2C in the IFM^15^. While previous data indicate that turnover of tendon collagen is low^14^,^44^, the IFM may contain a pool of more labile collagen. It is possible that more rapid turnover of collagen within the IFM provides a mechanism to prevent accumulation of damage within this region and thus maintain healthy tendon structure.

In addition, the reduction in the number of neopeptides identified with ageing in the IFM specifically may indicate reduced turnover of the IFM in aged tendons, which may have important implications for injury resistance. It has previously been shown that IFM stiffness increases with ageing, and the ability to resist cyclic loading decreases^16^,^17^. Taken together, these data suggest that reduced turnover of the IFM with increasing age may result in damage accumulation and alterations in IFM mechanical properties, increasing the risk of tendon injury in aged individuals^18^. Future studies should directly determine if alterations in the rate of IFM turnover are the cause of increased IFM stiffness.

Establishing the proteomic profile of distinct compartments of the tendon matrix provides an understanding of how differences in composition give rise to the distinct mechanical properties exhibited by the IFM and FM. This information is important to fully elucidate tendon structure-function relationships, and will help to guide the development of effective treatment options for tendinopathy. A full understanding of structure-function relationships within the relatively simple aligned fibre composite structure of tendon will also provide a basis for future studies investigating the interplay between structure and function in more complex fibrous soft tissues.

## Conclusions

Proteomic analysis has revealed for the first time how the protein profile differs between different tendon anatomical compartments and has identified changes that occur with ageing. The IFM, which plays an important mechanical role in the function of energy storing tendons, has a distinct proteomic profile from that of the fascicles. Further, the results suggest that the IFM may be turned over more rapidly than the FM, possibly providing a mechanism to prevent damage accumulation and maintain tendon health. While few proteins showed alterations in abundance with increasing age, there was a decrease in the number of neopeptides in the IFM with ageing, suggesting that the turnover of this matrix is decreased in aged individuals. This may result in accumulation of micro damage and a reduction in IFM mechanical integrity in aged tendon, contributing to age-related tendon injury.

## Methods

### Sample collection and preparation

The forelimbs of horses, euthanased for reasons other than tendon injury, were collected from a commercial equine abattoir, as a by-product of the agricultural industry. Specifically, the Animal (Scientific Procedures) Act 1986, Schedule 2, does not define collection from these sources as scientific procedures. Ethical approval was obtained for this study from the University of Liverpool Veterinary Research Ethics Committee (VREC214). The SDFTs were harvested from young (aged 4–7 years, n = 4) and old (aged 17–20 years, n = 4) horses. Horses reach skeletal maturity at 3 years old and are considered geriatric above the age of 15 years^45^. While it was not possible to obtain a full exercise history for the horses, none of the tendons had clinical or macroscopic evidence of tendon injury. For mass spectrometry analysis, samples (approximately 10 mm × 5 mm × 5 mm) were taken from the mid-metacarpal region of the tendon, embedded in Optimal Cutting Temperature solution (OCT), snap frozen in hexane cooled on dry ice, and stored at −80 °C. For Western blot analyses, longitudinal sections were taken from the mid-metacarpal region of the tendon, wrapped in tissue paper dampened in phosphate buffered saline (PBS) and tinfoil and stored at −80 °C.

### Mass Spectrometry Analyses

#### Laser Capture Microdissection

**Figure 5:**
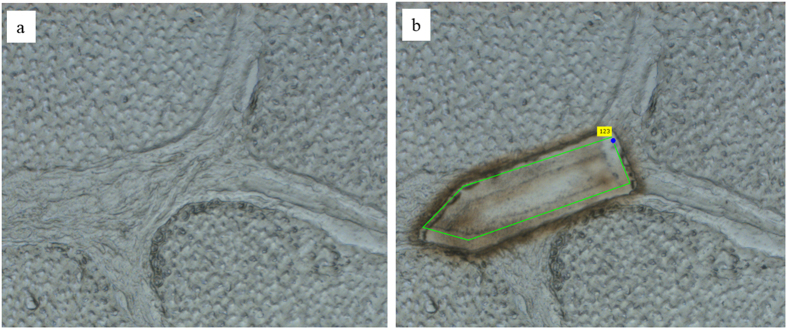
Laser capture microdissection was used to isolate regions of the IFM and FM. Transverse sections of the SDFT before (**a**) and after (**b**) a section of IFM has been collected.

Transverse cryosections were cut at a thickness of 8 μm from the OCT embedded tissue, adhered to membrane slides (1.0 PEN, Zeiss) and stored at −80 °C. Immediately prior to laser capture microdissection, slides were thawed, fixed in 70% ethanol, washed 6 times in ultrapure H_2_O to remove OCT and briefly fixed in 100% ethanol. Samples were allowed to dry before areas of FM and IFM were isolated using a laser ablation microscope (×10 objective, Zeiss PALM). To minimise tissue damage, laser power and focus were carefully optimised for each sample. Approximately 1 mm^2^ of IFM and FM was collected into adhesive caps (Zeiss) for each sample (Fig. 5). Samples were immediately frozen at −80 °C prior to protein extraction.

#### Protein Extraction

Samples were thawed and 50 μL of 0.1% (w/v) Rapigest (Waters, Manchester,UK) was added to the tubes and the tubes inverted to cover the tissue in the cap of the tube. Samples were incubated cap-side down for 30 min. at room temperature then heated at 60 °C for 60 min. Samples were trypsin digested and acidified as described previously^46^. C18 zip-tips were prepared with 3 membrane discs, following the Rubicon protocol: 40 μL wetting step with methanol followed by 70% (v/v) acetonitrile (ACN), followed by equilibration with 0.1% (v/v) trifluoroacetic acid (TFA). 20μL of sample was added and peptides were washed with 40 μL 0.1% (v/v) TFA. Peptides eluted with 50% (v/v) ACN/ 0.1% TFA (v/v) in ultrapure water. Samples were eluted into 1.5 mL low bind tubes and the volume reduced to approximately 3 μL by vacuum centrifugation. 12 μL of 3% (v/v) ACN/0.1% (v/v) TFA was added to each sample. Samples were centrifuged and transferred to total recovery vials for high resolution liquid chromatography tandem mass spectrometry (LC-MS/MS) analysis.

#### LC Separation

All peptide separations were carried out using an Ultimate 3000 Nano system (Dionex/Thermo Fisher Scientific) as described previously^46^, with 5 μl of sample injected.

#### Analyses on a Quadrupole-Orbitrap instrument

The Q Exactive (Thermo-Scientific, Waltham, USA) instrument was operated in data dependent positive electrospray ionisation mode to automatically switch between full scan MS and MS/MS acquisition. Survey full scan MS spectra (*m/z* 300–2000) were acquired in the Orbitrap with 70,000 resolution (*m/*z 200) after accumulation of ions to 1 × 10^6^ target value based on predictive automatic gain control values from the previous full scan. Dynamic exclusion was set to 20 s. The 10 most intense multiply charged ions (*z* ≥ 2) were sequentially isolated and fragmented in the octopole collision cell by higher energy collisional dissociation (HCD) with a fixed injection time of 100 ms and 35,000 resolution. Typical mass spectrometric conditions were as follows: spray voltage, 1.9 kV, no sheath or auxillary gas flow; heated capillary temperature, 250 °C; normalised HCD collision energy 30%. The MS/MS ion selection threshold was set to 1 × 10^4^ count and a 2Da isolation width was set.

#### Peptide Identification

Proteins within the IFM and FM were identified using Peaks® 7 PTM software (Version 6, Bioinformatics Solutions, Waterloo, Canada), searching against the UniHorse database, with a false discovery rate (FDR) of 1%, a minimum of 2 peptides per protein and a confidence score >20. The mass spectrometry proteomics data have been deposited to the ProteomeXchange Consortium (http://proteomecentral.proteomexchange.org) via the PRIDE partner repository^47^ with the dataset identifier PXD002979 and 10.6019/PXD002979.

#### Gene Ontology, Pathway Enrichment Analysis, and Protein Network Analysis

The horse proteome is incompletely annotated, therefore the gene symbols for each identified protein were searched in the Ensembl database for horse and converted to the gene symbol of the corresponding human orthologue. The resulting gene list was used for protein network analysis with the Search Tool for Retrieval of Interacting Genes/Proteins (STRING) version 9.1 48. The protein interaction maps were created by allowing for experimental evidence in addition to the predicted functional links: co-occurrence, co-expression, databases, and text-mining. Proteins were further characterised and classified using MatrisomeDB^49^ and Protein ANalysis THrough Evolutionary Relationships (PANTHER) Classification System^50^.

#### Label-free Peptide Quantification

For label-free quantification of proteins within the FM and IFM, the Thermo raw files of the acquired spectra from in-solution tryptic digests of FM and IFM samples were analysed by the Progenesis QI^TM^ software (Version 4, Waters, Manchester, UK) for label-free quantification as previously described^46^. Briefly, the top 5 spectra for each feature were exported from Progenesis QI^TM^ and utilized for peptide identification with a locally implemented Mascot server (Version 2.3.01), searching against the UniHorse database. Search parameters used were: 10 ppm peptide mass tolerance and 0.01 Da fragment mass tolerance; one missed cleavage allowed; fixed modification, carbamidomethylation; variable modification, methionine oxidation. Further analyses were performed in Peaks® 7, using the same modifications, to identify changes in protein abundance with ageing. Only unique peptides were used for quantification, and greater than 2 fold changes with FDR adjusted p values <0.05 were considered to be significant.

#### Ingenuity Pathway Analysis

Networks, functional analyses, and canonical pathways were generated through the use of ingenuity pathway analysis (IPA; Ingenuity Systems, Redwood City, CA, USA) on the list of differentially abundant proteins with value-adjusted P < 0.05 and ±2 fold regulation, quantified using ≥2 peptides. Gene symbols were used as identifiers. For network generation, datasets containing gene identifiers for differentially abundant proteins between the FM and IFM, and the corresponding fold change in abundance was uploaded into the application. These molecules were overlaid onto a global molecular network contained in the Ingenuity Knowledge Base. Networks of network-eligible molecules were then algorithmically generated based on their connectivity. The functional analysis identified the biological functions and diseases that were most significant to the dataset. A right-tailed Fisher’s exact test was used to calculate P values. Canonical pathways analysis identified the pathways from the IPA library of canonical pathways that were most significant to the dataset.

#### Neopeptide identification

To assess protein fragmentation, mass spectrometry data from the in-solution tryptic digests were analysed to identify neopeptides with at least one non-tryptic cleavage site. Neopeptides were identified by searches against the Unihorse database using Mascot as described previously^4^. Search parameters used were: enzyme, none; peptide mass tolerances 10 ppm; fragment mass tolerance of 0.01 Da, 1+, 2+, and 3+ ions; missed cleavages, 1. Modifications included were; fixed, carbamidomethyl cysteine and variable, oxidation of methionine, proline, and lysine. Neopeptides were only included if they were identified in 3 or more donors. Patterns of fragmentation were determined for collagens, proteoglycans and glycoproteins.

#### Determination of Cellularity

Longitudinal cryosections were cut at a thickness of 5 μm from the OCT embedded tissue. Sections were fixed in acetone for 10 min and washed in Tris buffered saline with 0.05% Triton-X. Cell nuclei were stained with DAPI and samples were imaged on a confocal microscope (Leica TCS SP2) using a ×40 oil objective. Brightfield images were used to identify the IFM and FM. 3 images were obtained from each section, and cell number and IFM and FM area was calculated (ImageJ, NIH, USA). Cell number was expressed per mm^2^. Data were tested for normality using D’Agostino & Pearson normality tests and significant differences determined using Dunn’s multiple comparison tests (Prism, GraphPad Software, Inc, La Jolla, USA).

### Western blotting validation of mass spectrometry data

#### Manual Microdissection

Thin longitudinal sections of the SDFT were viewed under a dissection microscope (Zeiss) and regions of IFM and FM were isolated using microdissection tools to tease apart the fascicles (Supplementary Fig. 2). Approximately 5 mg of IFM and FM were isolated from each sample and stored at −80 °C.

Proteins were extracted from the microdissected samples using Guanidine-HCl (Gnd-HCl). Samples were suspended in 15 volumes of 4M Gnd-HCl in 0.1M sodium acetate with protease inhibitors (cOmplete mini protease inhibitor cocktail tablets, Roche Diagnostics, Basel, Switzerland) and extracted for 24 hours at 4 °C with shaking. The samples were centrifuged at 4500 g for 10 min., the supernatant was removed to a clean tube and the remaining pellet was subjected to a further extraction overnight. Supernatant from the first and second extractions was combined, and Gnd-HCl was removed by precipitating twice in 95% ethanol in 0.1 M sodium acetate for 1 hour at −80 °C. Samples were resuspended in sodium dodecyl sulphate (SDS) buffer (25 mM Tris, 192 mM glycine, 0.1% SDS, pH 8.3; BioRad, Hercules, USA) and total protein content was determined using a Pierce™ bicinchoninic protein assay kit, according to the manufacturer’s instructions (Thermo Scientific).

To confirm the mass spectrometry data, the relative abundance of decorin, fibromodulin and COMP in the IFM and FM was determined using automated Western blotting (Wes Simple Western Analysis, ProteinSimple, San Jose, USA). Antibody details are shown in Supplementary Table 9. Simple Western analyses were performed according to the ProteinSimple user manual. Initial titrations were performed to optimise antibody and total protein concentration for each protein. In brief, Gnd-HCl extracted samples were mixed with a master mix (ProteinSimple) to give a final concentration of 0.004–0.2 mg/ml total protein, 1× sample buffer, 1x fluorescent molecular weight markers, and 40 mM DTT. Samples were heated at 95 °C for 5 min. Samples, blocking solution, primary antibodies, horseradish peroxidase-conjugated secondary antibodies, chemiluminescent substrate, and separation and stacking matrices were loaded into designated wells in a 384 well plate. After plate loading, fully automated electrophoresis and immunodetection took place in the capillary system. Proteins were separated by molecular weight at 375V for 25 min, and primary and secondary antibodies incubated for 30 minutes. All antibodies were diluted in a proprietary antibody diluent to the concentrations shown in Table 1 25,26,51. Chemiluminescence was captured by a charge-coupled device camera, and the digital image analysed using Compass software (ProteinSimple). The relative amount of each protein, relative to total protein content, was calculated based on peak area. A 29 kDa system control antibody (ProteinSimple) was spiked into each sample to provide within-capillary normalisation. Data were tested for normality using D’Agostino and Pearson normality tests (GraphPad Prism). Statistical significance was determined using t-tests or Mann Whitney tests, depending on whether the data were normally distributed (GraphPad Prism).

## Additional Information

**How to cite this article**: Thorpe, C. T. *et al*. Anatomical heterogeneity of tendon: Fascicular and interfascicular tendon compartments have distinct proteomic composition. *Sci. Rep.* **6**, 20455; doi: 10.1038/srep20455 (2016).

## Supplementary Material

Supplementary Information

Supplementary Tables

Supplementary Table 3

Supplementary Table 5

Supplementary Table 8

## Figures and Tables

**Table 1:** Classification of proteins in the IFM and FM of young and old tendons.

		Young IFM	Young FM	Old IFM	Old FM
ECM Proteins	**Total Protein Number**	**130**	**92**	**123**	**89**
	Collagens	16 (12.3%)	15 (16.3%)	14 (11.4%)	13 (14.6%)
	Glycoproteins	13 (10%)	8 (8.7%)	15 (12.2%)	8 (9.0%)
	Proteoglycans	8 (6.2%)	7 (7.6%)	9 (7.3%)	6 (6.7%)
	ECM regulators	4 (3.1%)	2 (2.2%)	1 (0.8%)	8 (9.0%)
	ECM-affiliated	3 (2.3%)	1 (1.1%)	4 (3.3%)	2 (2.2%)
	Secreted factors	2 (1.5%)	1 (1.1%)	2 (1.6%)	0
	**Total**	**47 (36.2%)**	**34 (37.0%)**	**45 (36.6%)**	**37 (41.6%)**
Non-ECM proteins	Cytoskeletal	36 (27.7%)	24 (26.1%)	41 (33.3%)	26 (29.2%)
	Histones	9 (6.9%)	22 (23.9%)	3 (2.4%)	4 (4.5%)
	Cell Membrane	5 (3.8%)	2 (2.2%)	4 (3.3%)	1 (1.1%)
	Enzymes	16 (12.3%)	2 (2.2%)	8 (6.7%)	6 (6.7%)
	Transcription/translation	7 (5.3%)	1 (1.1%)	2 (1.6%)	1 (1.1%)
	Other/Unknown	10 (7.7%)	5 (5.4%)	20 (16.2%)	10 (11.2%)
	**Total**	**83 (63.8%)**	**58 (63.0%)**	**78 (63.4%)**	**52 (58.4%)**

Proteins were classified using MatrisomeDB and PANTHER classification systems. Numbers in brackets indicate percentage of total protein number.

**Table 2:** Classification of differentially abundant proteins (identified using ≥ 2 peptides) in the IFM compared to the FM of young tendon.

		Higher in Young IFM	Higher in Young FM
ECM Proteins	**Total Protein Number**	**48**	**12**
	Collagens	1 (2.1%)	3 (25.0%)
	Glycoproteins	5 (10.4%)	5 (41.7%)
	Proteoglycans	1 (2.1%)	1 (8.3%)
	ECM regulators	0	1 (8.3%)
	ECM-affiliated	2 (4.2%)	0
	Secreted factors	0	0
	**Total**	**9 (18.8%)**	**10 (83.3%)**
Non-ECM proteins	Cytoskeletal	19 (39.6%)	1 (8.3%)
	Histones	6 (12.5%)	0
	Cell Membrane	0	0
	Enzymes	5 (10.4%)	0
	Transcription/translation	2 (4.2%)	0
	Other/Unknown	7 (14.6%)	1 (8.3%)
	**Total**	**39 (81.2%)**	**2 (16.6%)**

Numbers in brackets indicate percentage of total protein number. A full list of differentially abundant proteins is available in Supplementary Table 3.

**Table 3:** Number of neopeptides identified in each condition for a range of collagens and glycoproteins.

Protein	Young IFM			Young FM			Old IFM			Old FM		
	No. neo-peptides	No. specific to:		No. neo-peptides	No. specific to:		No. neo-peptides	No. specific to:		No. neo-peptides	No. specific to:	
		Region (IFM)	Age group (young)		Region (FM)	Age group (young)		Region (IFM)	Age group (old)		Region (FM)	Age group (old)
COMP	1	0	0	2	1	0	1	0	0	2	1	0
Decorin	2	0	0	2	0	0	1	0	0	2	0	0
Fibrillin-1	1	0	1	1	0	0	0	0	0	0	0	0
Fibronectin	0	0	0	0	0	0	0	0	0	0	0	0
TBSP-4	1	0	0	3	1	0	0	0	0	5	4	2
Col1A1	34	8	20	16	5	1	10	0	0	23	5	0
Col1A2	16	4	7	11	3	0	3	0	1	13	7	0
Col3A1	21	13	17	5	0	0	2	1	0	5	2	0
Col4A1	6	1	4	0	0	0	0	0	0	0	0	0
Col5A1	11	8	10	1	0	0	1	0	0	3	2	0
Col5A2	7	4	5	4	1	0	3	0	0	4	0	0
Col6A1	1	0	0	0	0	0	1	0	0	0	0	0
Col6A2	1	0	0	1	0	0	0	0	0	1	0	0
Col6A3	0	0	0	0	0	0	0	0	0	0	0	0
Col11A1	15	10	15	1	0	0	0	0	0	1	1	0
Col11A2	13	8	12	1	0	0	0	0	0	2	1	0
Col2A1 (Col11A3)	11	6	8	4	3	0	0	0	0	5	3	0
Col12A1	0	0	0	1	1	0	0	0	0	1	0	0
Total	**142**	**62**	**99**	**53**	**15**	**1**	**22**	**1**	**1**	**67**	**26**	**2**

The number of neopeptides that are only present in either IFM or FM, or with ageing, are also shown. Abbreviations: COMP, cartilage oligomeric matrix protein; TBS-4, thrombospondin-4.
