# Supplementary material for: Anatomical heterogeneity of tendon: Fascicular and interfascicular tendon compartments have distinct proteomic composition
Source: Sci Rep. 2016 Feb 4;6:20455. doi: 10.1038/srep20455 (PMC4740843; doi:10.1038/srep20455)
Supplement: Supplementary Information [file srep20455-s1.doc]

**Anatomical heterogeneity of tendon: Fascicular and interfascicular tendon compartments have distinct proteomic composition: Supplementary Information**

Chavaunne T Thorpe^1*^, Mandy J Peffers^2^, Deborah Simpson^3^, Elizabeth Halliwell^2^, Hazel R C Screen^1^, Peter D Clegg^2^.

^1^Institute of Bioengineering,

School of Engineering and Materials Science,

Queen Mary University of London,

Mile End Road,

London, E1 4NS, UK

^2^Department of Musculoskeletal Biology,

Institute of Ageing and Chronic Disease,

University of Liverpool,

Leahurst Campus,

Neston, CH64 7TE, UK

^3^Centre for Proteome Research,

Institute of Integrative Biology,

University of Liverpool,

Crown Street,

Liverpool, L69 7ZB, UK.

*Corresponding Author: [c.thorpe@qmul.ac.uk](mailto:c.thorpe@qmul.ac.uk)

**Supplementary Tables**

Supplementary Table 1. Details of peptides identified in the IFM (excel spreadsheet)

Supplementary Table 2. Details of peptides identified in the FM (excel spreadsheet)

Supplementary Table 3. Details of differentially abundant peptides between the IFM and FM in young tendon (excel spreadsheet)

Supplementary Table 4. Canonical pathways shown to be upregulated in the IFM compared to the FM in samples from young horses

Supplementary Table 5. Details of differentially abundant peptides between the IFM and FM in old tendon (excel spreadsheet)

Supplementary Table 6. Differentially abundant proteins with age in the IFM

Supplementary Table 7. Differentially abundant proteins with age in the FM

Supplementary Table 8. Details of neopeptides identified

Supplementary Table 9. Details of antibodies used for Western blotting validation of protein abundance

**Supplementary Figures**

Supplementary Figure 1. Protein-protein interaction map of proteins identified in the IFM (a) and FM (b). Proteins were input from the total dataset. Unconnected nodes were removed to enable clarity of the interactome. The total cluster was built with STRING allowing for experimentally verified and predicted protein-protein interactions at high confidence levels (0.7).

Supplementary Figure 2. A dissection microscope was used to visualise fascicles and IFM (a). Regions of IFM (b) and FM were isolated using microdissection tools.

| **Name** | **p-value** | **Ratio** |
| --- | --- | --- |
| ILK Signalling | 9.6E-11 | 10/186 (0.054) |
| Actin Cytoskeleton Signalling | 8.85E-09 | 9/217 (0.041) |
| Hepatic Fibrosis / Hepatic Stellate Cell Activation | 7.5E-08 | 8/197 (0.041) |
| Epithelial Adherens Junction Signalling | 1.69E-07 | 7/146 (0.048) |

Supplementary Table 4. Canonical pathways shown to be upregulated in the IFM compared to the FM in samples from young horses.

| **Highest condition** | **UniProt ID** | **Description** | **Classification** | **Peptide count** | **Mean fold change** | **ANOVA** |
| --- | --- | --- | --- | --- | --- | --- |
| **Young IFM** | P02538 | Keratin, type II cytoskeletal 6A | Cytoskeletal protein | 3 | 3.79 | 0.0007 |
|  | P08779 | Keratin, type I cytoskeletal 16 | Cytoskeletal protein | 3 | 3.47 | 0.009 |
|  | Q06828 | Fibromodulin | Proteoglycan | 8 | 3.02 | 0.01 |
| **Old IFM** | P62263 | 40S ribosomal protein S14 | Ribosomal protein | 1 | 61.88 | 0.002 |
|  | P50895 | Basal cell adhesion molecule | Immunoglobulin | 1 | 34.95 | 0.004 |
|  | P04040 | Catalase | Peroxidase | 1 | 13.70 | 0.01 |
|  | Q99972 | Myocilin | Structural protein receptor | 1 | 4.00 | 0.02 |

Supplementary Table 6. Differentially abundant proteins with age in the IFM. Proteins with a greater than 2 fold change in abundance and an FDR corrected p value < 0.05 were considered significant.

| **Highest condition** | **UniProt ID** | **Description** | **Classification** | **Peptide count** | **Mean fold change** | **ANOVA** |
| --- | --- | --- | --- | --- | --- | --- |
| **Young FM** | F6QAT0 | Collagen alpha-3(VI) chain | Collagen | 23 | 4.05 | 0.0006 |
|  | F6PVJ6 | Mimecan | Proteoglycan | 1 | 4.04 | 0.009 |
|  | Q06828 | Fibromodulin | Proteoglycan | 8 | 2.43 | 0.003 |
| **Old FM** | P05054 | Phospholipase A2 | Phospholipase | 3 | 10.87 | 0.002 |
|  | P02760 | Protein AMBP | Serine protease inhibitor | 1 | 6.38 | 0.01 |
|  | P31947 | 14-3-3 protein sigma | Chaperone | 4 | 5.82 | 0.04 |
|  | P62937 | Peptidylprolyl isomerase A | Isomerase | 1 | 3.15 | 0.04 |
|  | Q13310 | Polyadenylate-binding protein 4 | Transcription factor | 1 | 2.92 | 0.007 |
|  | P20774 | Plakophilin-3 | Cytoskeletal protein | 1 | 2.13 | 0.04 |

Supplementary Table 7. Differentially abundant proteins with age in the FM. Proteins with a greater than 2 fold change in abundance and an FDR corrected p value < 0.05 were considered significant.

| **Antibody** | **Species** | **Mono/ Polyclonal** | **Epitope recognised** | **Expected MW** | **Antibody concentration** | **Total protein (mg/ml)** | **Ref.** |
| --- | --- | --- | --- | --- | --- | --- | --- |
| Decorin (70.6) | Mouse | Monoclonal | Core protein | 50 KDa | 1:200 | 0.004 | Rees et al., 2000 |
| Fibromodulin (PR84) | Rabbit | Polyclonal | C-terminus (CGG)LRLASLIEI | 67 KDa | 1:25 | 0.2 | Roughley et al., 1996 |
| COMP | Rabbit | Polyclonal | Unknown | 110 KDa | 1:500 | 0.02 | Smith et al., 1997 |

Supplementary Table 9. Details of antibodies used for Western blotting validation of protein abundance

**Supplementary Figure 1.** **
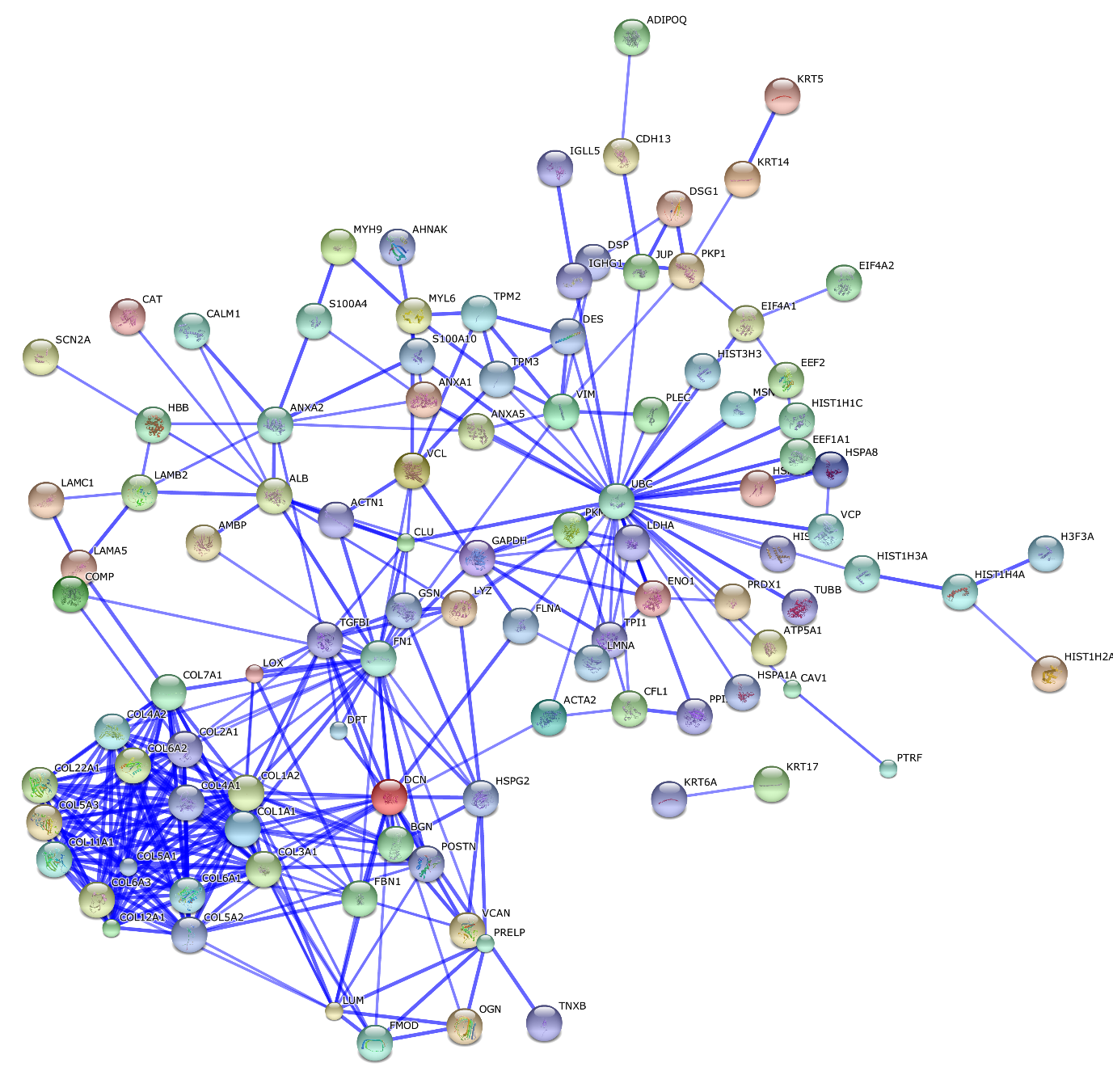
**

a

**
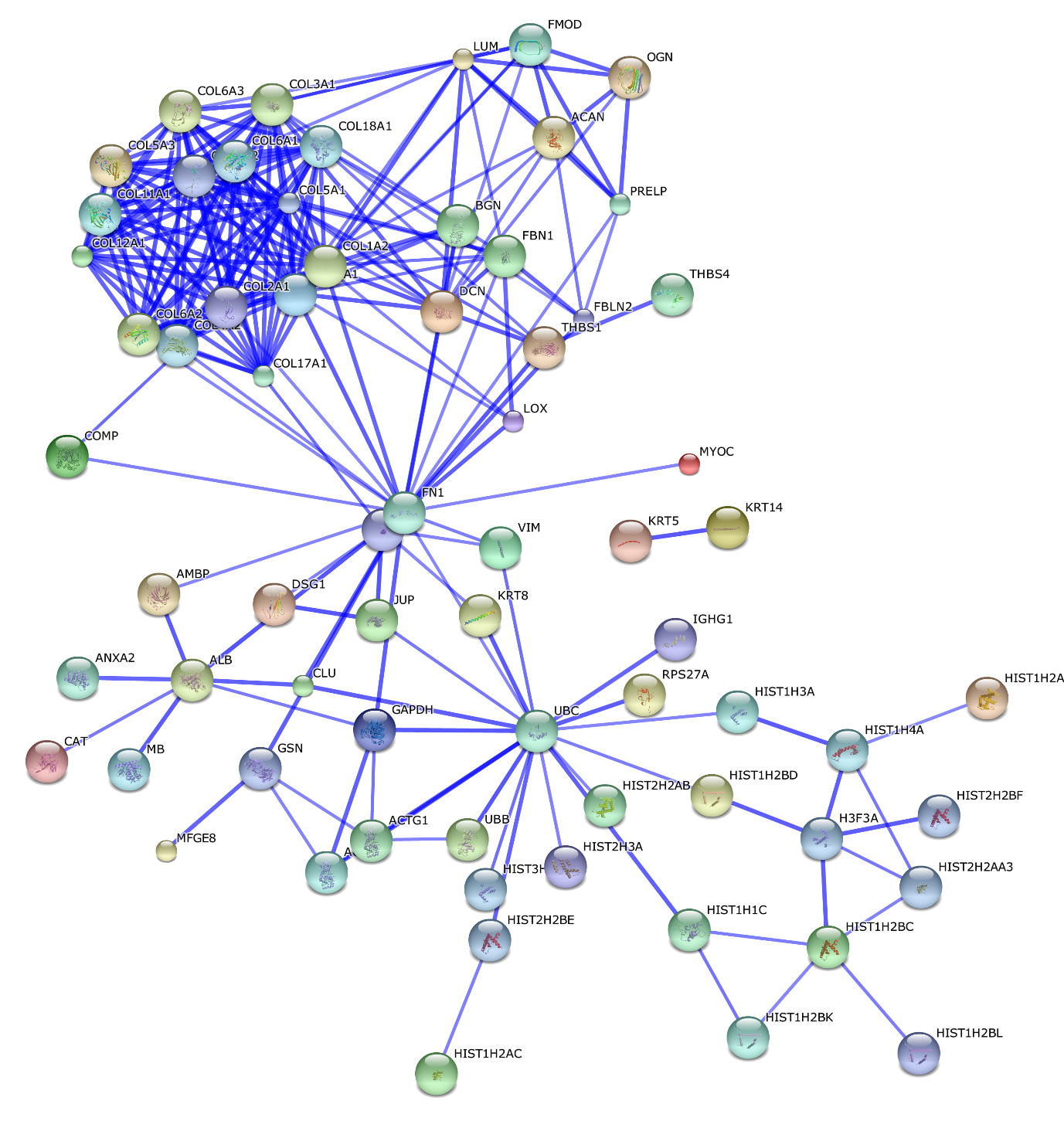
** Supplementary Figure 1. Protein-protein interaction map of proteins identified in the IFM (a) and FM (b). Proteins were input from the total dataset. Unconnected nodes were removed to enable clarity of the interactome. The total cluster was built with STRING allowing for experimentally verified and predicted protein-protein interactions at high confidence levels (0.7).

b

**Supplementary Figure 2**

**
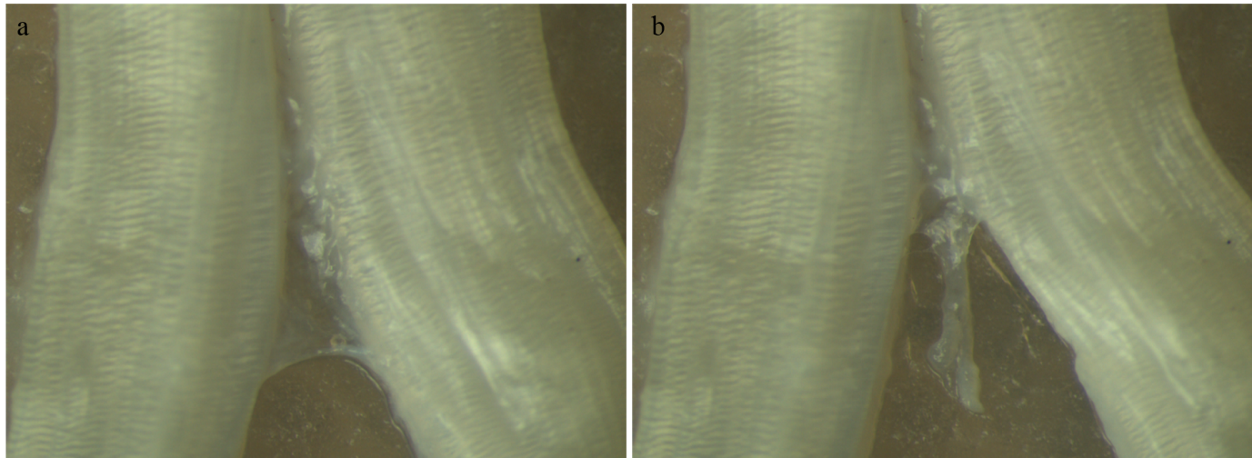
**

Supplementary Figure 2. A dissection microscope was used to visualise fascicles and IFM (a). Regions of IFM (b) and FM were isolated using microdissection tools.
